# Role of branched-chain amino acid metabolism in the pathogenesis of obesity and type 2 diabetes-related metabolic disturbances BCAA metabolism in type 2 diabetes

**DOI:** 10.1038/s41387-022-00213-3

**Published:** 2022-08-05

**Authors:** Froukje Vanweert, Patrick Schrauwen, Esther Phielix

**Affiliations:** Department of Nutrition and Movement Sciences, NUTRIM, School of Nutrition and Translational Research in Metabolism, Maastricht University Medical Center, Maastricht, The Netherlands

**Keywords:** Type 2 diabetes, Obesity

## Abstract

Branched-chain amino acid (BCAA) catabolism has been considered to have an emerging role in the pathogenesis of metabolic disturbances in obesity and type 2 diabetes (T2D). Several studies showed elevated plasma BCAA levels in humans with insulin resistance and patients with T2D, although the underlying reason is unknown. Dysfunctional BCAA catabolism could theoretically be an underlying factor. In vitro and animal work collectively show that modulation of the BCAA catabolic pathway alters key metabolic processes affecting glucose homeostasis, although an integrated understanding of tissue-specific BCAA catabolism remains largely unknown, especially in humans. Proof-of-concept studies in rodents -and to a lesser extent in humans – strongly suggest that enhancing BCAA catabolism improves glucose homeostasis in metabolic disorders, such as obesity and T2D. In this review, we discuss several hypothesized mechanistic links between BCAA catabolism and insulin resistance and overview current available tools to modulate BCAA catabolism in vivo. Furthermore, this review considers whether enhancing BCAA catabolism forms a potential future treatment strategy to promote metabolic health in insulin resistance and T2D.

## Introduction

Type 2 diabetes (T2D) is one of world’s most prevalent diseases, and is related to the epidemic of obesity [1]. Obesity can lead to the onset of T2D when pancreatic β-cells are no longer able to compensate higher insulin secretion for the reduced insulin sensitivity that often accompanies obesity [2]. Over the last decade, branched-chain amino acids (BCAA) catabolism has increasingly been considered to have an emerging role in the development of insulin resistance in people with obesity and T2D. In these individuals, BCAA levels are considerably elevated in plasma and tissues [3–9]. Furthermore, elevated BCAA levels in plasma strongly associate with insulin resistance in people with obesity and T2D [3, 4, 6–8, 10–13]. Although it is still unknown why these BCAA levels are elevated and why they associate with insulin resistance, a dysfunctional BCAA catabolism may be one of the underlying factors. This review aims to provide insight into the mechanisms behind elevated plasma BCAA levels in people with obesity and/or T2D and its role in the pathogenesis of insulin resistance. Furthermore, this review will overview pharmaceutical and alternative lifestyle intervention strategies in order to lower plasma BCAA levels and its effects on metabolic health.

## Why investigate BCAA levels?

Leucine, isoleucine and valine are grouped together as BCAA because they share a structural feature with a branched-side chain and common initiation steps of catabolism [14].

In general, BCAA play several important metabolic and physiological roles, aside from being considered as substrates for synthesis of proteins. Reports show that BCAA act as signaling molecules regulating metabolism of glucose, lipid, and protein [15]. In addition, BCAA levels play a key role in interorgan metabolic crosstalk and, therefore, dysregulation of BCAA catabolism may play a significant role in several metabolic diseases [16].

Several studies showed that plasma BCAA levels in overweight and obese humans with insulin resistance [3–7] and patients with T2D [8, 9] were elevated compared to healthy individuals. Recently, in an observational study, we confirmed this finding and showed that plasma BCAA levels were elevated in patients with T2D compared to age- and BMI-matched controls without having T2D [13]. Some [17–19], but not all studies [20, 21] found elevated plasma BCAA levels to be associated with increased risk of T2D and suggest that BCAA levels in plasma may predict future diabetes [17].

It has repeatedly been reported that the accumulation of plasma BCAA levels strongly associate with insulin resistance in obesity and T2D [3, 4, 6–8, 10–13]. Similarly, a short-term intravenous infusion with amino acids in young, human volunteers induced temporary insulin resistance [22]. However, as a mixture of amino acids were infused, it cannot be deduced from this study whether the BCAA per se are responsible for the development of insulin resistance. So far, there are no reports investigating whether particularly a raise of BCAA plasma levels in humans induces insulin resistance. Therefore, the underlying mechanisms of elevated BCAA plasma levels on insulin-stimulated glucose uptake in humans remain largely unknown.

## Why are plasma BCAA levels elevated with insulin resistance?

BCAA homeostasis and levels in plasma are defined by BCAA appearance and disappearance, affected by several processes. Processes contributing to BCAA appearance in the blood include protein breakdown in tissues (a process which is inhibited by insulin), food intake and gut microbial synthesis. The major processes involved in disappearance of BCAA are protein synthesis, excretion and BCAA catabolism [4, 23]. As a result, an interplay between these mechanisms defines the levels of BCAA in plasma, and therefore multifactorial causes could underlie the elevated BCAA plasma levels seen in people with insulin resistance and patients with T2D.

### Effect of insulin on protein breakdown and BCAA catabolism

Insulin is known to be one of the most important regulators of carbohydrate, fat and protein metabolism. Protein metabolism, or more specifically, protein turnover, is defined by the balance between protein synthesis and protein breakdown [24]. During periods of steady state, the rate of protein synthesis equals the rate of protein breakdown. Both insulin as well as BCAA concentrations affects protein turnover in muscle [25], adipose tissue [26] and liver [27].

The effect of insulin on leucine flux has been investigated in humans with use of an intravenous infusion of insulin combined with [1-^13^C] or [1-^14^C]-leucine tracer [28–30]. An intravenous insulin infusion in people without diabetes provoked a decline in the leucine flux due to a reduction in protein breakdown, without an effect on protein synthesis [28–30]. The activation of protein kinase B (Akt) in response to insulin by the insulin receptor (IRS-1) induces phosphorylation of the Forkhead box class (FOXO) transcription, and indirectly activate mTOR, which seems to be responsible for the inhibited muscle protein breakdown via [31–35].

In humans with insulin resistance, the effect of insulin on reducing muscle protein breakdown is blunted causing increased muscle wasting [36], as is confirmed in rodent models [37–40]. BCAA are reported to activate the mTOR pathway [41] and stimulate protein synthesis in muscle of humans. However, the inhibitory effect of insulin on protein breakdown occurs independently of the levels of circulating plasma BCAA [42–44]. Normally, insulin’s inhibitory action on protein breakdown in muscle tissue [45–47] results in lower amino acid concentrations in plasma [42, 48, 49], with the most marked decline seen for BCAA [50–53]. The effect of insulin on BCAA plasma levels has been investigated for the first time in patients with type 1 diabetes [54, 55] and results showed that the withdrawal of insulin treatment was associated with a substantial increase in circulating BCAA concentrations, as confirmed by others [58, 59]. We recently confirmed the strong insulin-suppressive effect on BCAA levels in plasma during a euglycemic hyperinsulinemic clamp in healthy, insulin sensitive people with obesity, however, this insulin-suppressive effect was blunted in people with obesity, diagnosed with non-alcoholic fatty liver (NAFL) and/or T2D [56]. Also others found less efficient BCAA reduction upon insulin infusion in obese humans with insulin resistance [57–59]. The suggestion that increased BCAA levels could merely be a consequence of impaired insulin action is in accordance with the results from a recent mendelian randomization study [60], showing that insulin resistance drives higher plasma BCAA levels [60, 61]. In contrast, a large-scale human genetic study by Lotta et al. pointed towards a causal role of diminished BCAA catabolism underlying insulin resistance [62], which is described below.

### Diet and microbiome

BCAA cannot be synthesized by humans and are therefore essential dietary components that must originate from ingested food [63]. In addition, gut microbiota is able to produce and degrade BCAA [64].

Major dietary sources of BCAA include milk, red meat, poultry, and high fat dairy products [65, 66]. BCAA make up almost 20% of dietary protein [63]. Since the Western diet is characterized by high fat and protein intake [3], one could assume that dietary intake of protein may contribute to changes in plasma BCAA levels. Indeed, evidence suggests that consumption of dietary protein increases the risk of diabetes and insulin resistance [3, 66, 67]. Newgard et al. [3] reported that individuals with obesity and insulin resistance consumed more protein compared to lean individuals. Since in the individuals with obesity and insulin resistance BCAA levels in plasma were increased, this data matches the assumption that higher protein intake leads to increase of BCAA in plasma [3]. However, in these studies only intake of total protein had been assessed, and not the BCAA consumption. In contrast, others found that BCAA levels were elevated in individuals with insulin resistance compared to healthy participants, despite equal rates of protein intake. Furthermore, a weak correlation was found between BCAA dietary intake and plasma BCAA levels [4, 19, 65]. McCormack et al. found that plasma BCAA levels, but not dietary BCAA intake, was associated with obesity and insulin resistance [19].

Besides direct dietary intake, BCAA can also be metabolized by the gut microbiome [68–71]. More specifically, a recent study by Pedersen et al. [70] showed that a gut microbiome having a higher potential for biosynthesis of BCAA and reduced number of inward bacterial transporters for these amino acids were associated with increased levels of BCAA in plasma [70]. Interestingly, increased potential for BCAA biosynthesis and reduced potential for bacterial BCAA uptake are both linked with insulin resistance [70]. Above all, it has been reported that circulating BCAA levels were increased in mice following transplantation of stool derived from individuals with insulin resistance [64]. This data indicates that microbiota indeed contributes to changes in BCAA plasma levels, in which altered gut microbiota could be another underlying cause of elevated BCAA levels in individuals with insulin resistance.

### BCAA catabolism

#### BCAA catabolism in health

**Fig. 1:**
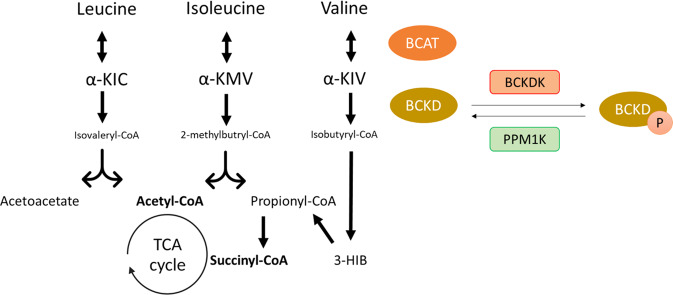
Schematic overview of BCAA catabolism. BCAT branched-chain amino acid transaminase, BCKD branched-chain keto acid dehydrogenase, α-KIC α-ketoisocaproate, α-KMV α-keto-methylvalerate, α-KIV α-ketoisovalerate, 3-HIB 3-hydroxyisobutyrate, BCKDK BCKDK kinase, PPM1K BCKDK phosphatase. Adapted from Neinast et al. [73].

Catabolism of all three BCAA, leucine, isoleucine and valine, is located inside the mitochondria, in which the first two steps are common for all BCAA (Fig. 1) [72, 73]. The first reaction is the reversible transamination catalyzed by the branched-chain amino acid aminotransferases (BCAT) to form branched-chain α-keto acids (BCKA): α-ketoisocaproate (α-KIC), α-keto-B-methylvalerate (α-KMV), and α-ketoisovalerate (α-KIV), respectively formed out of leucine, isoleucine and valine [74]. The second step is the irreversible oxidative decarboxylation by the branched-chain α -keto acid dehydrogenase (BCKD) complex, the rate-limiting enzyme of this pathway [75]. BCKD comprising three catalytic components (E1, E2 and E3) is regulated by a phosphorylation-dephosphorylation catalyzing process, whereby a specific kinase (BCKDK) is responsible for inactivation and a phosphatase (PPM1K) for activation of this complex [76, 77], both regulated by nutrient status and BCAA levels itself [78–80]. It has been reported that phosphorylation occurs in the E1 component of the BCKD complex, whereas dephosphorylation reaction interacts with both the E1 and E2 domain [77, 81–83]. Ultimately, the CoA compounds formed by the BCKD-complex are further metabolized to acetyl-CoA and succinyl-CoA, which are incorporated into the tricarboxylic acid (TCA) cycle [84]. TCA cycle fueling also occurs via the alanine cycle (or termed Cahill cycle), which is tightly linked to BCAA catabolism. The alanine cycle involves series of reactions in which amino groups and carbons from skeletal muscle are transported to the liver [85]. In short, in skeletal muscle, the reaction of BCAA to BCKA yields glutamate which then combines with pyruvate to generate alanine [86]. Alanine is released by skeletal muscle and taken up by the liver [87, 88], where it forms an important source for gluconeogenesis [89]. The glucose produced by the liver is shuttled into the circulation, taken up by muscle cells [87], and consequently converted back to glutamate, entering the TCA cycle via α-ketoglutarate [86].

Tissue-specific BCAA metabolism has been investigated in rodent models. Neinast et al. investigated whole-body BCAA catabolism in mice using in vivo isotopic tracing and found that most tissues actively oxidize BCAA, with the largest contribution likely in skeletal muscle and liver [84]. Other rodent studies showed that BCAT activity, the enzyme responsible for the BCAA transamination step, is relatively low in hepatocytes [90]. Moreover, unlike other amino acids, BCAA circumvent first-pass metabolism in the liver [84], and are primarily transaminated to BCKA in extra-hepatic tissues since BCAT is mainly expressed in muscle, kidney and heart tissue in rodents [63, 91, 92]. Next, BCKA are released back into the circulation and undergo oxidation by the BCKD complex in the liver [76]. Accordingly, it has been assumed that the liver of rodents has the highest BCKD activity [93], however, BCKD is also expressed in white adipose tissue (WAT) although to a lesser extent [75, 76].

Information on tissue-specific BCAA oxidation in humans is, however, very limited. In one study, enzymatic activities of BCAT and BCKD were evaluated in several human-derived tissues and showed large differences compared to the results observed in rodent tissues [76]. Thus, Suryawan et al. [76] reported that both skeletal muscle and liver in humans are key tissues involved in BCAA catabolism and express BCAT and BCKD, with the highest expression in muscle, which was also found by others [85]. Furthermore, human heart [94–96] and adipose tissue [75, 81, 97–102] depend on BCAA oxidative capacity as well.

#### BCAA catabolism in obesity and T2D

Since the first two steps of BCAA catabolism are common for all three BCAA, a reduced BCAA catabolic flux in one of these steps forms a plausible explanation underlying the rise in plasma BCAA levels of obese insulin resistant individuals with and without T2D. Indeed, several studies points towards diminished or altered function of the key enzymes involved in BCAA catabolism [23, 75, 103–105]. This has been confirmed in rodent studies showing that increased levels of BCAA in plasma are the result of reduced expression of BCAT [75, 106] or lower BCKD complex activity, via either increased expression of BCKDK [75, 84, 107, 108] or suppression of PPM1K [80, 103, 109, 110]. Animal models of obesity and T2D as well show affected BCAA catabolism [75, 111, 112]: tissue-specific expression of BCAA-catabolic enzymes are shown to be dysregulated [23, 27, 79, 108, 113–121] especially in adipose tissue [75, 122] and liver [75, 113]. Moreover, decreased BCAA catabolism in WAT is assumed to be a contributor to increased plasma levels of BCAA as seen in obesity and insulin resistance [75, 81, 97–102, 104]. The capacity of WAT to modulate circulating BCAA levels has been confirmed by Herman et al. [98], who demonstrated that transplantation of normal WAT into transgenic mice with defective peripheral BCAA catabolism reduced circulating BCAA levels.

Although only limited knowledge derives from human studies, collecting evidence supports the hypothesis that dysfunctional BCAA catabolism could underlie a rise in BCAA plasma levels. For instance, in patients with maple syrup urine disease (MSUD), an inborn error of metabolism caused by loss-of-function mutation in components of the BCKD complex [123–126] or its regulatory phosphatase, PPM1K [127], BCAA levels in plasma are found to be elevated. Others confirmed that altered activity of BCAT or the BCKD complex, at least in muscle and liver, plays a role in plasma BCAA levels [25, 62, 93, 117, 128, 129]. Reduced expression levels of BCAT were found in skeletal muscle of insulin resistant patients with T2D, which could explain the observed elevated BCAA plasma levels [117]. Also expression of PPM1K in skeletal muscle of people with T2D failed to increase in contrast to healthy controls during in oral glucose challenge, which could indicate dysregulation of the BCAA pathway [62]. Indeed, gene expression studies revealed downregulation in multiple steps of the BCAA catabolic pathway in skeletal muscle of individuals with insulin resistance [25, 129] and patients with T2D [62]. In addition, individuals with obesity and/or T2D were shown to have a marked decrease in BCKD protein content in liver biopsies when compared to the non-obese control group [93]. In human liver cells, mutation or deletion of PPM1K resulted in elevated BCAA levels [128].

The BCAA catabolic pathway has also been shown to be downregulated in WAT of people with obesity [99]. The idea that BCKD in WAT contributes to changes in BCAA levels in humans is supported by the fact that BCAA levels in plasma significantly decreased after bariatric surgery [75, 130], while BCKD expression in WAT increased [75]. Together, these results demonstrate the capacity of WAT to modulate circulating BCAA levels. WAT is, however, suggested to be responsible for less than 5% of whole-body BCAA oxidation [84], meaning that the increase in plasma BCAA levels must have additional origins [131].

Others have suggested that reduced BCAA oxidation in adipose tissue and liver may induce BCAA overflow to skeletal muscle, driving its BCAA oxidation there [23, 84, 131–133]. Since skeletal muscle has a high capacity to oxidize BCAA, it could be postulated that muscle functions as the metabolic sink for impaired BCAA oxidation in adipose tissue and liver [132]. Interestingly, a recent study using a heavy isotope steady-state infusion of BCAA, showed a shift in BCAA oxidation from adipose tissue and liver toward skeletal muscle in obese, insulin resistant mice [84], consistent with the finding that BCKD enzyme activity in liver and adipose tissue is downregulated in animals with obese/insulin-resistant or diabetic states [75, 103, 111, 112, 134–138]. This was also confirmed by She et al. who found that BCKD activity was decreased in adipose tissue.

Recently, we reported that in vivo whole-body leucine oxidation rates were significantly lower in patients with T2D compared to control participants with similar age and BMI [13]. Previously, no differences were reported between FDR and matched controls [139] nor between obese and control participants [140]. As leucine, valine and isoleucine share the same oxidation route via the BCKD complex, one could assume that in vivo 1-^13^C leucine tracer kinetics represent the total BCAA pool [141–143]. Nevertheless, it would be of interest to measure the oxidation rates of the three individual BCAA (i.e., with 1-^13^C leucine, 1-^13^C isoleucine, and 1-^13^C isoleucine), which has never been investigated in humans. Furthermore, as BCAA and BCAA-derived catabolites has mostly been investigated in plasma, levels in human peripheral tissues would give more insight into tissue-specific BCAA catabolism. These considerations highlight the need for future research to investigate whether tissue-specific BCAA catabolic defects occur in individuals with obesity, insulin-resistance or T2D individuals.

## How do plamsa BCAA levels link to insulin resistance?

**Fig. 2:**
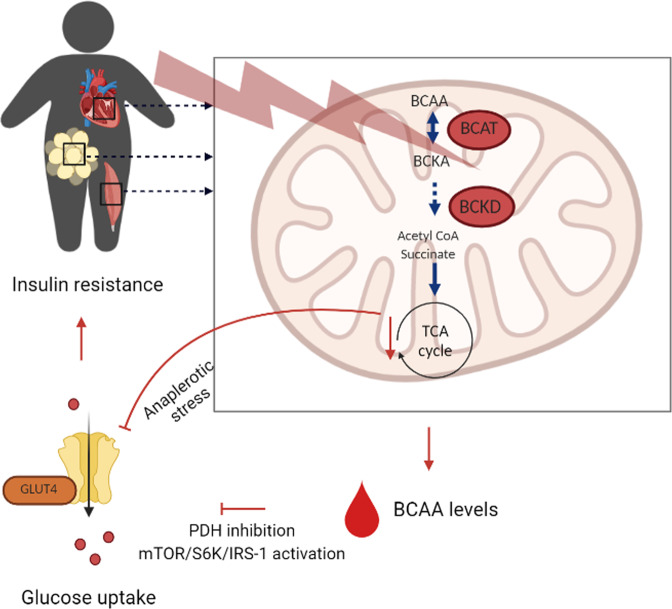
Schematic overview of mechanisms linking BCAA catabolism with insulin resistance. BCAA branched-chain amino acids, mTOR mammalian target of rapamycin complex, S6K ribosomal S6 kinase, IRS-1 insulin receptor substrate-1, PDH pyruvate dehydrogenase complex, GLUT4 glucose transporter type 4.

As already mentioned, several reports have been suggested that increased BCAA levels could merely be a consequence of impaired insulin [60, 61], however, evidence indicates that plasma BCAAs act as signaling molecules and contribute to the development of insulin resistance in humans [3, 5, 22, 25, 144–147]. Several mechanisms have been hypothesized explaining how plasma BCAA levels contribute to insulin resistance, which are overviewed in Fig. 2 and discussed in the following paragraphs.

### Dysfunctional mitochondrial BCAA catabolism

We as well as others have repeatedly reported that people with insulin resistance and patients with T2D feature low muscle mitochondrial oxidative capacity [13, 148, 149]. The end products of BCAA catabolism inside the mitochondria, succinyl-CoA and acetyl-CoA, enter the TCA cycle and are important anaplerotic substrates fueling the TCA cycle. Defects in BCAA-catabolic enzymes may cause so-called anaplerotic stress and underlie low mitochondrial respiratory rates resulting in disturbed glucose and fat oxidation seen in this population [25, 62], which has been supported by in vitro studies [150–153]. In humans, it has been hypothesized that individuals with impaired or incomplete BCAA metabolism are susceptible to develop insulin resistance [23], in which anaplerotic stress originating from reduced BCAA-derived carbon flux to TCA cycle intermediates is an important underlying factor [23, 138, 154–158]. Additional studies investigating this concept, are however warranted.

Dysfunctional mitochondrial BCAA catabolism may explain the accumulation of a number of BCAA-catabolic metabolites in plasma in insulin-resistant people with obesity or T2D, including BCAA-derived acylcarnitines (C3 and C5), 3-hydroxyisobutyrate (3-HIB) and 2-hydroxbutyric acid (2-HB) and 2-ketobutyric acid (2-KB) [3, 10, 25, 81, 133, 137, 159, 160], which can have toxic effects on cellular function. It has been shown that acylcarnitines can cause mitochondrial dysfunction [3, 23, 47, 133, 161–164]. Furthermore, several studies link defective BCAA catabolism and consequently accumulation of toxic metabolites to increased lipotoxicity [109, 127, 128, 165, 166] and insulin resistance [3, 23, 47, 133, 161–164]. 3-hydroxyisobutyrate (3-HIB), a catabolic intermediate of valine, can exit the mitochondrion via the covalent binding to CoA [146]. Several reports have indicated an elevation of 3-HIB in plasma of people with insulin resistance [146, 167]. In addition, comprehensive metabolic profiling found that 2-HB and 2-KB, both catabolites of methionine/threonine metabolism, are elevated in individuals with reduced insulin sensitivity [168]. Moreover, in individuals with impaired glucose tolerance, plasma levels of 2-HB associate with hyperglycemia and insulin sensitivity and are an early marker for insulin resistance and risk for future T2D [169–171]. Interestingly, since 2-HB can be produced from and converted back into 2-KB, and 2-KB is an BCKD substrate, the increase in these metabolites may reflect impaired BCAA catabolism [172].

To summarize, dysfunctional mitochondrial BCAA catabolism in several tissues may cause anaplerotic stress thereby dysregulating glucose and fat oxidation (Fig. 2). Accumulation of either toxic BCAA-intermediates may exacerbate mitochondrial dysfunction, linked to impaired glucose homeostasis and insulin resistance.

### Elevated BCAA levels hamper insulin signaling pathways

#### mTOR/S6K pathway

Both insulin and BCAA are known to stimulate the activity of mammalian target of rapamycin (mTOR), although the mechanisms for their action is not completely understood [173]. In normal conditions, insulin mediates phosphorylation of IRS-1, which in turn activates the phosphatidylinositol 3-kinase (PI3K)/Akt pathway [174]. Akt regulates glucose transport via the phosphorylation of Akt substrate of 160 kDa (AS160) to trigger GLUT4 translocation from intracellular site to the surface of the cell [23, 175–177]. In addition, Akt is able to activate mTOR via phosphorylation of tuberous sclerosis complex 1/2 (TSC 1/2) leading to degradation of Ras homolog enriched in brain (Rheb) [174], which alleviates the inhibition of mTOR [175]. To summarize, insulin is able to activate mTOR via the PI3K-Akt signaling pathway [178].

It has been suggested that increased BCAA levels in plasma or tissue also activate the mTOR pathway, although independently of TSC regulation [179, 180]. Elevated BCAA levels could lead to persistent activation of mTOR followed by serine phosphorylation of IRS-1 via S6 kinase (p70S6K). Phosphorylation of IRS-1 prevents further Akt-signaling leading to diminished glucose transport and consequently insulin resistance [181, 182]. Therefore, chronic accumulation of plasma BCAA levels could impede with the insulin signaling via activation of the mTOR/p70S6K pathway [181–184] with leucine as most potent mTOR activator [180].

BCAA-induced activation of the mTOR/p70S6K pathway has been shown by multiple rodent studies [3, 133, 146, 181, 182, 185, 186] and cell culture experiments [187–189]. In addition, in vivo and in vitro BCAA deprivation in mice reduced the activation of the mTOR pathway and increased pAkt in liver and muscle, resulting in improved insulin sensitivity [190–192]. Interestingly, Newgard et al. reported that dietary BCAA-induced mTOR activation only occurred in the presence of a high fat load [3, 104]. Moreover, mTOR-stimulated pAkt activation in muscle with the consequent development of insulin resistance, solely occurred when BCAA were supplemented in combination with a high-fat diet, and not upon BCAA supplementation combined with chow [3, 104]. Overall, collecting data in preclinical models support the notion that elevated BCAA availability - especially under high fat conditions - plays a key role in the development of insulin resistance, mediated by downregulation of PI3K-Akt signaling pathway and hyperactivation of the mTOR/p70S6K pathway.

Evidence for a role of BCAA in mTOR signaling and insulin resistance in humans is scarce. A short-term infusion of a mixture of amino acids, including BCAA, activated mTOR paralleled by reduced peripheral insulin sensitivity in humans [181, 184]. In addition, Weickert et al. [193] showed that a 6-week high-protein diet enriched with leucine and isoleucine, induced insulin resistance with increased p70S6K levels observed in adipose tissue [193]. Although these results show that BCAA-induced mTOR activation play a role in the development of insulin resistance in humans, normalized BCAA plasma levels which occurred after gastric bypass surgery, did not result in reduced mTOR activation [159], although insulin resistance improved substantially in these patients. The excessive weight loss in the latter study therefore seems to be the driving factor underlying improved insulin sensitivity, and not the change in BCAA plasma levels per se.

#### Inhibition of PDH

Pyruvate dehydrogenase complex (PDH) is the rate-limiting enzyme involved in glucose oxidation [194], linking glycolysis to the TCA cycle by transferring pyruvate into acetyl-coenzyme A (CoA) [94]. A common manifestation in obese individuals with insulin resistance is the inability to shift from fatty acid oxidation in the fasted state to glucose oxidation in the fed state, also called metabolic inflexibility [195]. This fatty acid-induced suppression of glucose oxidation as well glucose disposal can be explained by the model of Randle et al. [196]: by-products of fatty acid oxidation, such as acetyl-CoA, NADH and ATP, act as potent allosteric inhibitors of glycolysis and PDH [197]. Several studies in animals reported that accumulation of BCAA and its derived metabolites can also directly inhibit PDH activity, at least in liver [153, 198] and heart [94, 152, 199], resulting in a marked decrease in glucose uptake and oxidation. Moreover, animal studies show that dysfunctional BCAA oxidation result in accumulation of BCAA in cardiac tissue and forms a hallmark in cardiovascular disease [95, 200]. A mouse model with impaired BCAA oxidation revealed that the chronic accumulation of BCAA in heart tissue suppressed glucose metabolism [94]. More specifically, high levels of BCAA selectively disrupted mitochondrial pyruvate (end product of glucose oxidation) utilization through inhibition of PDH activity. It has long been established that PDH activity is a key determinant for insulin resistance of the heart [201, 202], in which BCAA may play a pivotal role. This link has not been investigated in humans, however, one study demonstrated that BCAA concentrations accumulate in failing heart tissue as a resultant of a coordinated decrease in BCAA oxidative genes [95], and was associated with impaired cardiac insulin signaling. However, whether BCAA-inhibited PDH activity played a role, was not investigated. In addition, one study showed that supplementing BCAA during exercise as well as during the recovery period resulted in increased plasma glucose levels due to reduced glucose uptake in the leg in the recovery period [203]. The authors suggest that the oxidation of supplemented BCAA resulted in increased BCAA-oxidative derived acetyl-CoA concentrations thereby inhibiting PDH activity, however, the elevated BCAA levels could as well be responsible for reduced pyruvate utilization.

Although there is evidence that elevated BCAA levels hamper insulin signaling pathways, it remains still unclear whether elevated BCAA levels are a cause or rather a consequence of insulin resistance. Future research, specifically cohort studies, could provide more information about causality between BCAA levels and insulin resistance.

## Effective strategies to lower BCAA levels

### Pharmaceutical strategies

#### BT2

**Fig. 3:**
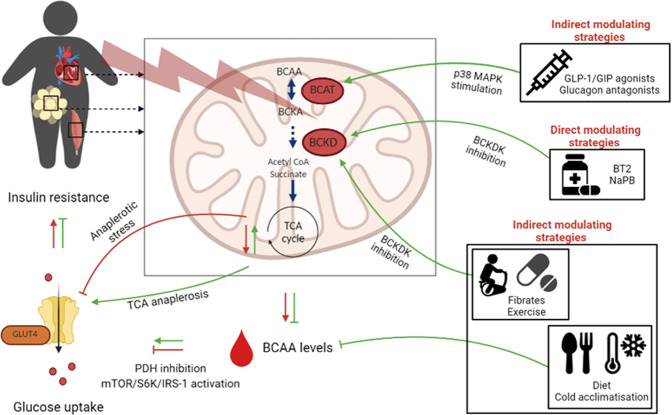
Schematic overview of pharmaceutical and alternative strategies and their hypothesized way of action to boost BCAA oxidation and lower BCAA levels. BCAA branched-chain amino acids, mTOR mammalian target of rapamycin complex, S6K ribosomal S6 kinase, IRS-1 insulin receptor substrate-1, PDH pyruvate dehydrogenase complex, GLUT4 glucose transporter type 4, BT2 3,6-dichlorobenzo(b)thiopene-2-carboxylic acid, NaPB sodium phenylbutyrate, GLP-1 GCR-like peptide-1, GIP glucose-dependent insulinotropic polypeptide.

A compound called 3,6-dichlorobenzo(b)thiopene-2-carboxylic acid (BT2) is a small-molecule inhibitor of BCKDK and accelerates the BCAA catabolic pathway via increased activation of the BCKD complex (Fig. 3) [95, 204]. Its working mechanism has been confirmed in obese and diabetes mice models, who report accelerated BCAA catabolism in skeletal muscle [84, 200], liver, heart and adipose tissue [105, 107]. In these models, the administration of BT2 resulted in lower plasma BCAA levels, improved insulin sensitivity and hyperinsulinemia, and reduced hepatic fat levels [105, 107]. Together, these results demonstrate that BT2 is effective to restore BCAA catabolic activity in various tissues alleviating the BCAA catabolic defect, and thus improving insulin sensitivity, irrespective of the site.

Furthermore, several studies administered BT2 in mice with heart failure [110, 205, 206], and collectively show that dysfunctional BCAA catabolism plays a pivotal role in the development of cardiac dysfunction. Results show that BT2-induced accelerated cardiac BCAA catabolism in failing hearts decreased cardiac BCAA levels, with beneficial effects on heart tissue remodeling, improved cardiac insulin sensitivity and function [110, 200, 205, 206]. The mechanisms underlying the cardiometabolic protective effects observed in these studies remain to be elucidated, however, results point out that restoring dysfunctional BCAA catabolism optimizes substrate use and attenuates mitochondrial function [110, 205, 206]. Interestingly, some studies show that the beneficial effects of BT2 on improved glucose metabolism were exerted by reduced mTOR activity and/or via a reduction in the formation of BCAA-derived toxic metabolites [110, 205]. To conclude, BT2 is a pharmacological agent which directly modulate BCAA catabolism via activating BCKD activity. As BT2 is not suitable for human use, so far, effects of pharmacologically modulating BCAA catabolism on the human heart and other tissues, as well on glucose homeostasis has not been investigated in humans.

#### NaPB

Sodium phenylbutyrate (NaPB) is a commonly used medication for the treatment of patients with urea cycle disorders [207]. NaPB is an aromatic fatty acid that is converted in vivo by β-oxidation into phenylacetate followed by conjugation with glutamine to form phenylacetylglutamine, which is excreted in the urine [208]. Via this mechanism NaPB act as an ammonia scavenger in patients with urea cycle disorders [209]. Interestingly, it has been demonstrated in mice [210] and human cells [208] that NaPB, as BT2, also directly enhance BCAA catabolism through stimulation of the BCKD complex by preventing the phosphorylation of BCKDK (Fig. 3). Holecek et al. [211] showed that in vitro and in vivo administration of NaPB resulted in augmented BCAA catabolism resulting in reduced BCAA levels in plasma and muscle [211]. In another in vitro study in mice, NaPB treatment resulted in lower BCAA concentrations paralleled by improved insulin-stimulated glucose uptake [189, 212] via an improved insulin signaling in skeletal muscle cells [189]. This result was confirmed in a diabetic mouse model showing substantial improved glucose metabolism upon NaPB treatment [213]. These data postulate that NaPB-induced lowering of BCAA levels alleviate the inhibition of insulin signaling leading to an improved glucose uptake, in which skeletal muscle plays an important role.

Although limited research has been performed in humans, some studies show that NaPB lowers BCAA levels in patients with urea cycle disorders, patients with MSUD and healthy subjects [207, 208, 214–217]. In a study with male people with overweight or obesity, NaPB administration was effective in partially improving lipid-induced insulin resistance, although circulating plasma BCAA levels were not measured [218]. As previously done in mouse skeletal muscle cells [189], it would be of interest to study effects of NaPB administration on insulin signaling and glucose uptake in primary human muscle cells, to acquire missing physiological insights on the metabolic consequences of modulating BCAA catabolism in humans.

#### Fibrates

Fibrate is a class of drugs widely used to treat dyslipidaemia by reducing cholesterol and triglyceride levels, decreasing the risk for the development of cardiovascular diseases [219, 220]. Fibrate mechanism of action includes activation of peroxisome proliferator-activated receptor alpha (PPARα), a transcriptional factor of genes involved in fatty acid oxidation [219, 220]. The major adverse effect of the clinical use of fibrates is the development of myopathy [221–223], however, the pathogenesis of fibrate-induced myopathy is still unclear.

In rodents, several studies showed that fibrate treatment decreased BCAA and BCKA plasma levels [224–226] as well in skeletal muscle and liver tissue [227]. Fibrates inhibit gene expression of the BCKDK in the liver (Fig. 3) [225, 226, 228–231], an effect which was not found in skeletal muscle [228]. This could imply that fibrates enhance BCAA catabolism specifically in the liver.

Interestingly, it has been shown that fibrate treatment improved insulin sensitivity in patients with T2D, although the underlying mechanisms were not investigated [232–234]. Fibrate treatment decreased the activation of the mTOR/p70S6K pathway in rats [226], as well lowered BCAA plasma levels in humans [235]. Whether the fibrate-induced improvement in insulin sensitivity is attributable to improved BCAA catabolism, lower BCAA levels and/or decreased activation of the mTOR-pathway, cannot be deduced from these studies.

#### Novel T2D therapies targeting incretin and glucagon receptors

In recent years, new therapies targeting receptors including GCR-like peptide-1 (GLP-1), glucose-dependent insulinotropic polypeptide (GIP) and glucagon have been developed. Tirzepatide, a dual GIP and GLP-1 agonist and potential new glucose-lowering medication for patients with T2D, has been shown to improve hyperglycemia [236]. Obese insulin resistant mouse models feature improved glycaemic control in the presence of reduced BCAA and BCKA plasma levels upon Trizepatide treatment [237]. The observed effects were accompanied by an increased expression of BCAT via the p38-MAPK pathway particularly in BAT (Fig. 3) [237]. Interestingly, in humans, Tirzepatide treatment reduced BCAA, BCKA and other BCAA-derived metabolites in plasma, including 3-HIB and 2-HB, previously shown to associate with insulin resistance and T2D [238]. Together, tirzepatide may alter expression of genes regulating BCAA catabolism explaining these results [238]. Also, antagonizing the glucagon receptors has shown to be effective in improving insulin sensitivity in models of diabetes and obesity [239]. In failing heart, inhibition of the glucagon receptor improved insulin-stimulated glucose oxidation and enhanced cardiac function, which were attributable to an improved BCAA catabolism via the p38-MAPK pathway [240]. Although these findings suggests that T2D treatment targeting receptors as GLP-1, GIP and glucagon may activate BCAA catabolism, future studies will be required to investigate if and how activated BCAA catabolism helps to improve glycaemic control upon this treatment in individuals with insulin resistance and T2D.

### Alternative strategies

#### Physical activity and exercise

Generally, it has been assumed that amino acids do not contribute substantially to energy supply during endurance exercise training [241]. In contrast, others suggest that this assumption may underestimate the role of proteins and that endurance exercise may result in promotion of amino acid catabolism in general, and especially the oxidation of BCAA [242]. To provide energy, endurance training promotes the transamination of BCAA to BCKA [75], which are further metabolized into acyl-coenzymes which can enter the TCA cycle [84]. Indeed, it is well established that endurance exercise training in rodents [243] and combined endurance and resistance training in humans with overweight [244] decreased plasma BCAA levels and toxic intermediates of BCAA catabolism, such as acylcarnitines. Consistent with this finding, a recognized effect of endurance exercise training is an accelerated BCAA catabolism represented by an increased BCKD activity [245]. More specifically, it has been found that BCKD is activated due to decreased phosphorylation by BCKD kinase (Fig. 3) [246–250]. Several exercise intervention studies in rats found that BCKD complex was activated in skeletal muscle [78, 251], as well as in liver [248, 249]. The mechanisms responsible for activating these enzymes are not fully understood. One report demonstrated that inactivity potently downregulated expression of BCAA metabolic genes in mice and vice versa that expression of BCAA metabolic enzymes were upregulated in response to endurance exercise training [25]. Contrarily, others suggest that the relative short exercise training sessions, as performed in the beforementioned studies, could not underlie altered gene expression or phosphorylation status of the kinase and that other mechanisms are possibly involved [248, 252].

Recently, we found that levels of BCAA were lower in more active individuals compared to less active individuals [56], which is in line with another observational study showing an association between high physical activity level and low plasma BCAA levels [253]. Nevertheless, 12-week combined endurance and resistance-exercise training in people with obesity did not result in decreased plasma BCAA levels [56]. Although prolonged intense exercise has been shown to increase the activity of the BCKD complex in skeletal muscle of trained, healthy individuals [254], this effect might be blunted in people with insulin resistance. Controversy does exist on the effect of exercise on BCAA catabolism. Howarth et al. [255] showed that a single bout of endurance exercise increased BCKD kinase content in human skeletal muscle, which was associated with a training-induced decrease in BCKD activity, although Poortmans et al. did not find a change in plasma BCAA levels [256]. The inconsistent responses of the different studies could be explained by different work load, duration of physical activity and exercise training, and individuals’ training status. In addition, changes in plasma BCAA levels upon exercise are not a good reflection of BCAA catabolism since exercise influence protein turnover, and therefore also BCAA levels. Exercise training studies combined with stable isotope would elucidate the impact of exercise on BCAA catabolism. The question, however, remains if improved BCAA catabolism is involved in the improvement in metabolic health after physical activity and exercise.

#### Dietary restriction of BCAA

As mentioned before, diet may contribute to the elevation of BCAA as observed in humans, and therefore diet intervention could potentially help to improve BCAA metabolism. Indeed, it has been shown that restricting dietary BCAA restores metabolic health, including lower adiposity and improved insulin sensitivity in obese rodents [257–259]. The positive metabolic effects were independent of alterations in BCKD activity [260] suggesting that low protein diets restrict plasma BCAA levels thereby alleviating its inhibitory effect on glucose uptake.

In humans, BCAA dietary restriction studies are limited since feasibility is a challenge: interpretation can be limited in case nitrogen and caloric content is different between intervention arms, and therefore any reported effects cannot be asserted as solely due to BCAA restriction. It has been shown, that BCAA levels decreased after a weight loss program, but was not related to changes in BCAA intake [10].

One study reported only modest changes in fasting BCAA levels, associated with an increase in insulin sensitivity upon short-term dietary restriction in healthy individuals [261]. Patients with T2D are characterized by higher plasma BCAA levels compared to healthy controls and therefore probably may benefit more from a BCAA restricted diet. Indeed, short-term dietary reduction of BCAA was effective in decreasing BCAA levels coinciding with improved postprandial insulin sensitivity and gut microbiome composition in patients with T2D [262]. Although, reports showed in vivo and in vitro that lowering BCAA levels alleviates the inhibition of the insulin signaling pathway by decreasing mTOR/S6K1 signaling resulting in increased insulin sensitivity [191, 262], when and how BCAA restriction influences metabolic health, particularly glucose homeostasis, remains unclear. Long-term studies in humans are needed to evaluate the safety and the metabolic efficiency in individuals with obesity and insulin resistance.

#### Cold acclimatization

Several rodent reports noted that cold exposure significantly decreases plasma BCAA levels, possibly by an increased BCAA uptake and oxidation merely located in BAT [263–266]. Consistent with their findings, it was recently reported that BCAA are actively utilized in BAT mitochondria for UCP1-mediated thermogenesis upon cold exposure in mice [266]. In turn, impaired capacity to take up BCAA and defective BCAA catabolism in BAT results in impaired BCAA clearance and thermogenesis leading to impairments in lipid and glucose metabolism [266, 267]. Thus, besides glucose and fatty acids, BCAA are likely to be important energy substrates in BAT during cold exposure, however, the relationship of BCAA metabolism to thermogenesis is still unclear.

Also in humans, Yoneshiro et al. [266] observed that cold exposure for 2 h preferentially decreased BCAA plasma levels in participants with high BAT activity, suggesting a potential link between BAT and BCAA metabolism. Surprisingly, muscle mass showed no correlation with cold-induced changes in BCAA levels although skeletal muscle is a major organ that utilizes BCAA [266]. Feasibility

To summarize, catabolism and levels of BCAA can be modulated by several pharmaceutical and alternative strategies, although their mechanisms are not completely known in humans. Further research would be needed to study feasibility and optimization for alternative strategies. As a side note, BT2 and NaPB are the only interventions able to directly target the BCAA catabolic defect to improve glucose homeostasis. Other pharmaceutical and alternative interventions, known to improve metabolic health, have also shown to influence BCAA catabolism and levels, however, it has not yet been investigated whether this improved metabolic health is attributable to change in BCAA catabolism and levels.

## Conclusion

Dysregulation of BCAA catabolism is closely related to obesity- and T2D related metabolic disturbances since BCAA levels plays a key role in interorgan metabolic crosstalk. Findings from animal and human studies provided evidence that dysfunctional BCAA catabolism in several tissues could be a plausible explanation for the elevated plasma BCAA levels seen in obesity and T2D, however, huge knowledge gaps exist in tissue-specific BCAA catabolism in humans. Insulin resistance can occur via dysfunctional BCAA catabolism or BCAA levels acting as signaling molecules hampering the insulin signaling pathways. Therefore, exploring intervention strategies to increase BCAA oxidation and/or lower BCAA levels is important to investigate whether this could be a new potential strategy in the treatment of metabolic diseases, including obesity and T2D.
